# Cytotoxicity and genotoxicity of nano - and microparticulate copper oxide: role of solubility and intracellular bioavailability

**DOI:** 10.1186/1743-8977-11-10

**Published:** 2014-02-13

**Authors:** Annetta Semisch, Julia Ohle, Barbara Witt, Andrea Hartwig

**Affiliations:** 1 Department of Food Chemistry and Toxicology, Karlsruhe Institute of Technology (KIT), Institute for Applied Biosciences, Adenauerring 20a, Karlsruhe 76131, Germany

**Keywords:** Copper oxide nanoparticles, Copper oxide microparticles, Copper chloride, Bioavailability, Intracellular distribution, Cytotoxicity, Genotoxicity, Poly(ADP-ribosyl)ation, Apoptosis

## Abstract

**Background:**

Nano- or microscale copper oxide particles (CuO NP, CuO MP) are increasingly applied as catalysts or antimicrobial additives. This increases the risk of adverse health effects, since copper ions are cytotoxic under overload conditions.

**Methods:**

The extra- and intracellular bioavailability of CuO NP and CuO MP were explored. In addition, different endpoints related to cytotoxicity as well as direct and indirect genotoxicity of the copper oxides and copper chloride (CuCl_2_) were compared.

**Results:**

Comprehensively characterized CuO NP and CuO MP were analysed regarding their copper ion release in model fluids. In all media investigated, CuO NP released far more copper ions than CuO MP, with most pronounced dissolution in artificial lysosomal fluid. CuO NP and CuCl_2_ caused a pronounced and dose dependent decrease of colony forming ability (CFA) in A549 and HeLa S3 cells, whereas CuO MP exerted no cytotoxicity at concentrations up to 50 μg/mL. Cell death induced by CuO NP was at least in part due to apoptosis, as determined by subdiploid DNA as well as via translocation of the apoptosis inducing factor (AIF) into the cell nucleus. Similarly, only CuO NP induced significant amounts of DNA strand breaks in HeLa S3 cells, whereas all three compounds elevated the level of H_2_O_2_-induced DNA strand breaks. Finally, all copper compounds diminished the H_2_O_2_-induced poly(ADP-ribosyl)ation, catalysed predominantly by poly(ADP-ribose)polymerase-1 (PARP-1); here, again, CuO NP exerted the strongest effect. Copper derived from CuO NP, CuO MP and CuCl_2_ accumulated in the soluble cytoplasmic and nuclear fractions of A549 cells, yielding similar concentrations in the cytoplasm but highest concentrations in the nucleus in case of CuO NP.

**Conclusions:**

The results support the high cytotoxicity of CuO NP and CuCl_2_ and the missing cytotoxicity of CuO MP under the conditions applied. For these differences in cytotoxicity, extracellular copper ion levels due to dissolution of particles as well as differences in physicochemical properties of the particles like surface area may be of major relevance. Regarding direct and indirect genotoxicity, especially the high copper content in the cell nucleus derived after cell treatment with CuO NP appears to be decisive.

## Background

The transition metal copper is an essential trace element and a catalytic cofactor in more than 30 enzymes [1,2]. Under physiological conditions, the cellular copper homeostasis is tightly regulated [3]. However, under overload conditions copper is potentially toxic: Due to its redox activity copper catalyzes Fenton-type reactions, generating the highly reactive hydroxyl radical, which may damage cellular components like proteins, nucleic acids or membrane lipids [4]. Furthermore, due to its high affinity towards thiols, copper may bind to and/or oxidize redox-sensitive amino acids like cysteines, thereby destroying redox-sensitive protein structures. This may also include proteins or enzymes with zinc-binding domains of proteins or enzymes involved in maintaining genomic stability, such as several DNA repair and DNA damage signalling enzymes like PARP-1 [5,6].

Potential conditions for copper overload are supranutritional intake, e.g., due to food supplementation, genetic disorders or inhalation of copper containing fumes or aerosols. With respect to the latter, particulate copper oxides like synthesized CuO NP or CuO MP are increasingly applied as catalysts, antimicrobial additives or in pigment production [7-10]. Nevertheless, appropriate toxicological examinations of risks and benefits of nano-sized materials are still rare. Different to water soluble metal compounds, where the toxicologically relevant interactions depend on the chemical properties of the respective metal ions, the physicochemical characteristics of particles and their interactions with cells additionally affect biological outcomes and underlying mechanisms [11,12]. Relevant features are size, morphology, specific surface area and crystallinity, elemental composition as well as solubility in biologically relevant media [13].

The particle characteristics described above will also determine the uptake, intracellular bioavailability and thus the potential toxicity of the respective compounds. While copper ions enter the cell via transporters, nano- and microsized particles may be internalized by endocytosis [14,15], which in case of CuO NP has been visualized and confirmed, mainly using transmission electron microscopy (TEM) [16-18]. Once inside the cell, toxicologically relevant reactions will also depend on the release of ions of the respective material in different cellular compartments including the lysosomes and subsequent concentrations reached in the cytoplasm and in the cell nucleus.

Cu-based nanoparticles have been shown to be particularly cyto- and genotoxic, both, when compared to other metal-based nanoparticles or when compared to copper-based microsize particles [19,20]. Also, with respect to genotoxicity, CuO NP induced DNA damage to a greater extent than CuO MP in the comet assay [17,20,21]. Additionally, incubation with CuO NP increased the extent of chromosomal damage as determined by micronuclei formation [22-24].

This raises the question on the underlying mechanisms. In principle, toxic reactions could be due to the interaction of the particles with the plasma membrane or with intracellular components. Furthermore, the deliberation of copper ions due to extracellular or intracellular dissolution of the particles could contribute to the observed effects. The aim of the present study was to systematically investigate and compare the toxic and genotoxic potential of well characterized CuO NP, CuO MP and CuCl_2_ in A549 and HeLa S3 cells. Special emphasis was given to the extracellular and intracellular bioavailability of copper ions. We determined the dissolution of CuO NP and CuO MP in model fluids like artificial alveolar fluid (AAF), Dulbecco’s Modified Eagle Medium with serum (DMEM/FCS) or without serum (DMEM) as well as in artificial lysosomal fluid (ALF). In a further approach, we determined the bioavailability and intracellular distribution of copper ions in cells. In order to relate these findings to biological effects we examined cytotoxicity, apoptosis, induction of DNA strand breaks and micronuclei formation. Furthermore, we determined the impact on poly(ADP-ribosyl)ation, since this reaction involved in DNA damage signalling has been shown previously to be inhibited by water soluble CuCl_2_[6]. Our results demonstrate that especially the increase in nuclear copper concentrations will determine the direct and indirect genotoxicity of the respective copper compounds.

## Results

### Particle characterization

**Figure 1:**
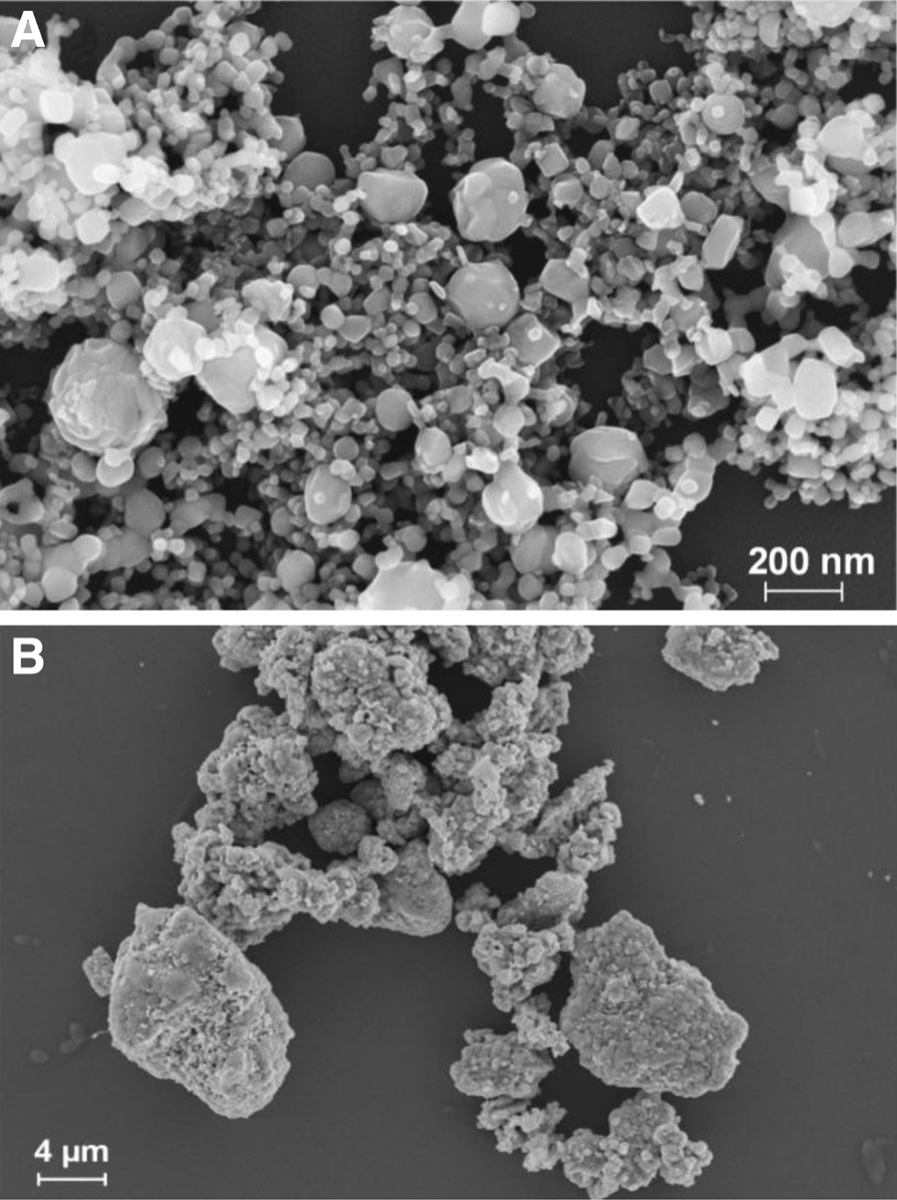
**SEM images CuO NP and CuO MP.** Images of **(A)** CuO NP (Magnification 150000 ×) and **(B)** CuO MP (Magnification 6500 ×) were taken at 10 kV acceleration voltage.

First, the particles were characterized with respect to chemical composition, crystallinity, morphology, endotoxin contamination as well as size, surface area, pH and Zeta potential (ZP), also in cell culture media. Scanning electron microscopy revealed an approximately spherical and smooth surface of CuO NP. They exhibited a broad size distribution ranging from approximately 20 nm to around 200 nm (Figure 1A). CuO MP appeared rough and irregularly shaped. They were hardly distinguishable as individual particles and seemed to be aggregated or agglomerated consisting of smaller submicron particles. To discriminate between agglomerates and aggregates, particles were treated with an ultrasound tip for 60s. Since no breakdown of particles was observed (data not shown), they were most likely aggregated. Size reached in some cases more than 10 μm (Figure 1B). The specific surface area, as determined by Brunauer-Emmett-Teller (BET) analysis, was around 23 times larger in case of CuO NP (17.23 m^2^/g) when compared to CuO MP (0.74 m^2^/g). Calculated average diameters, assuming uniform spherical particles, were 55 nm (CuO NP) and 1289 nm (CuO MP). These values confirm data provided by the manufacturer (CuO NP: ≤ 50 nm, CuO MP: < 5 μm). Both substances did not alter the pH of the cell culture medium DMEM/FCS, were free of endotoxines, of crystalline form and exhibited a purity of > 99.8% as determined by Inductively coupled plasma mass spectrometry (ICP-MS), analysis of the oxygen content and Energy-dispersive X-ray spectroscopy (EDX) (data not shown). To also define particle characteristics under cell culture conditions, the Dynamic light scattering (DLS) and ZP were determined in ultrapure water (H_2_O), DMEM and DMEM/FCS using the Zetasizer Nano ZS (Malvern). As a prerequisite to DLS and ZP measurements, the viscosity, diffraction index, density and the dielectricity constants of DMEM and DMEM/ FCS were determined. The viscosity in the presence of salts (DMEM, 1.09 mPas) and serum (DMEM/FCS, 1.15 mPas) was slightly increased while the other characteristics resembled those observed in H_2_O (listed in Materials and methods).

Suspended in H_2_O, DMEM or DMEM/FCS CuO NP revealed a size distribution centered around a hydrodynamic radius of 488 nm, 205 nm and 146 nm, respectively, indicating the formation of larger agglomerates in the absence of buffer components and serum proteins. The ZP was -14.4 mV (DMEM), - 13.1 mV (DMEM/FCS) and -4.5 mV (H_2_O). Due to rapid sedimentation, the CuO MP could not be analysed by DLS.

### Solubility in model fluids

**Figure 2:**
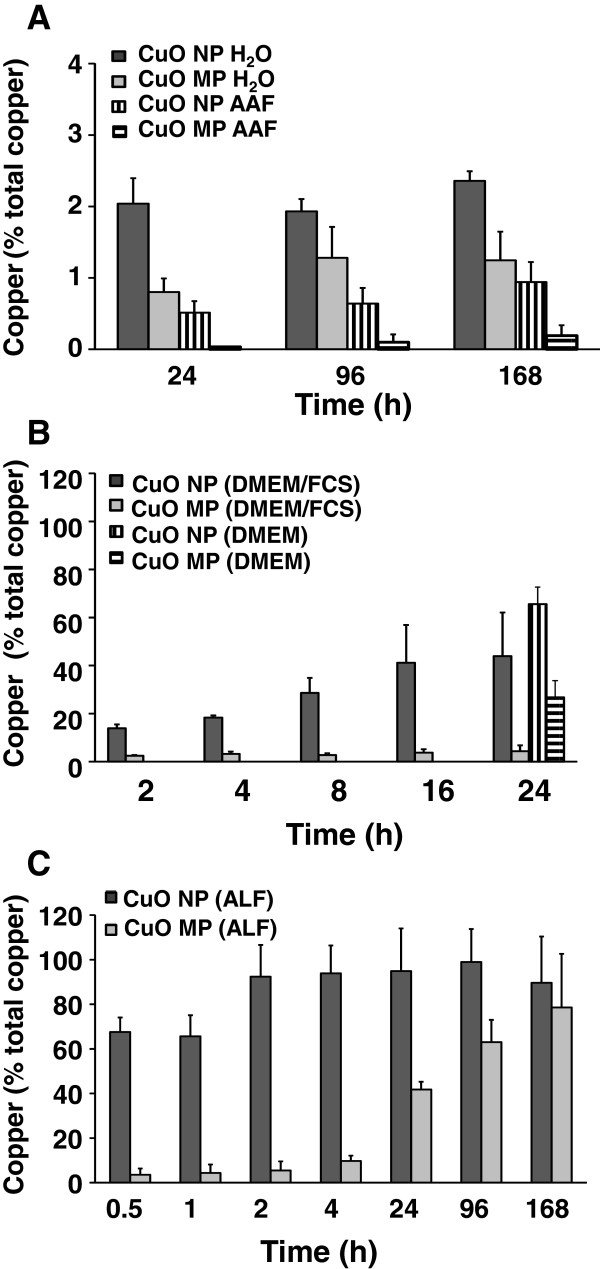
**Dissolution of CuO NP and CuO MP in different media.** CuO NP and CuO MP were incubated in **(A)** H_2_O or AAF for 24, 96 or 168 h at 37°C, **(B)** in DMEM/FCS for 2, 4, 8, 16 or 24 h or in DMEM without FCS for 24 h under cell culture conditions (37°C, 5% CO_2_, 100% humidity) or **(C)** in ALF for 0.5, 1, 2, 4, 24, 96 or 168 h at 37°C. Remaining particles were removed from the solution by multiple centrifugation as described in Materials and methods. The copper content was quantified by GF-AAS. Shown are mean values of 3 independent determinations + SD. 100% total copper refer to 39.95 mg/L in case of 50 μg/mL CuO. Please note the different scales of the Y-axis.

Next, the solubility of the particles was investigated in several model fluids. Even though the results are not directly transferable to biological fluids where for example the formation of protein coronas appear to be of major importance, the solubility in different media provide important information on the ratio between particulate and soluble fractions of the respective particles in the experimental system and also some hints on their intracellular fate. Thus, the dissolution of CuO MP and CuO NP was quantified in H_2_O, phosphate buffered saline (PBS), DMEM, DMEM/FCS and AAF. Since after endocytosis the particles are located in lysosomes, copper ion release was additionally determined in ALF (pH 4.5). After the specified incubation times the supernatants were repeatedly centrifuged and the concentrations of released copper were quantified by graphite furnace atomic absorption spectroscopy (GF-AAS). In H_2_O, PBS (data not shown) and AAF, dissolution for both particles types was below 2.4%, with CuO NP releasing more copper ions than CuO MP (Figure 2A). Nevertheless, dissolution of CuO NP was highly accelerated in a time-dependent manner in cell culture medium (DMEM) supplemented with FCS: Here, after 2 h, 14% of the copper content were solubilized, reaching 44% after 24 h. In contrast, copper ion release from CuO MP remained low with 4% dissolved copper after 24 h incubation. In DMEM in the absence of FCS, the solubility was higher for both particle types: 66% of copper ions were released from CuO NP and 27% from CuO MP after 24 h (Figure 2B). The accelerated solubility of CuO NP as compared to CuO MP was even more pronounced in an acidic environment. Thus, in ALF, 68% of the CuO NP was already solubilized after 30 min, and after 2 h dissolution was almost complete. In contrast, CuO MP revealed only 10% dissolution after 4 h and about 80% solubilization was reached only after 168 h (Figure 2C).

### Cytotoxicity

**Figure 3:**
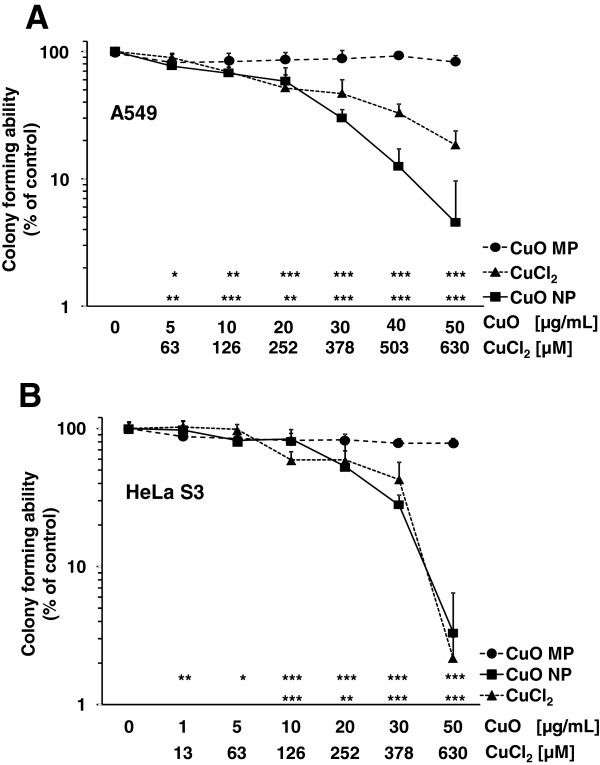
**Cytotoxicity of CuO NP, CuO MP and CuCl**_**2**_**.** Cytotoxicity was determined by colony forming ability (CFA). Logarithmically growing **(A)** A549 or **(B)** HeLa S3 cells were treated with CuO NP, CuO MP or CuCl_2_ for 24 h, trypsinized, counted and reseeded. Shown are mean values of at least 9 determinations + SD. Statistically different from control cells: *p < 0.05, **p < 0.01, ***p < 0.001 as determined by unifactorial analysis of variance (ANOVA) followed by Dunnett’s T3 test. 1 μg/mL are equal to 0.2 μg/cm^2^ and 50 μg/mL CuO are equal to 630 μM Cu^2+^ in case of complete dissolution.

To compare the cytotoxicity and to define appropriate incubation conditions for the subsequent experiments, the colony forming abilities (CFA) of A549 and HeLa S3 cells after 24 h incubation with CuO NP, CuO MP or CuCl_2_ were investigated. This approach was chosen since nanoparticles and metals ions may interfere with frequently applied dye-based toxicity assays. In case of copper, CuO NP and CuCl_2_ were observed to interfere with the reduction of [2-(2-methoxy-4-nitrophenyl)-3-(4-nitrophenyl)-5-(2,4-disulfophenyl)-2H-tetrazolium, monosodium salt] (WST-8) to the corresponding formazan [25]. In A549 cells, strongest dose-dependent cytotoxicity was seen in case of CuO NP, followed by CuCl_2_. Thus, after 24 h incubation, a significant decrease was observed for CuO NP at 5 μg/mL, with a residual viability below 10% CFA at the highest concentration of 50 μg/mL. Based on the copper content, cytotoxicity of CuCl_2_ was similar at low concentrations, but less toxicity was seen at higher concentrations. Surprisingly, CuO MP were not cytotoxic over the whole concentration range (Figure 3A). Similar effects were evident in HeLa S3 cells: While again CuO MP were not cytotoxic, in this case CuO NP and CuCl_2_ led to comparable reduction in CFA, based on the total copper content of the two compounds (Figure 3B). Due to the high cytotoxicity of CuO NP and CuCl_2_, the subsequent experiments were conducted with particle concentrations up to 20 μg/mL CuO or 252 μM CuCl_2_, respectively, except for investigations on apoptosis and the induction of micronuclei.

### Apoptosis

To investigate whether the cytotoxicity is due to apoptosis, three different parameters were analysed in A549 cells, namely the translocation of the apoptosis inducing factor (AIF) into the cell nucleus, the impact of the copper compounds on caspase 3 and 7 activities as well as the accumulation of subdiploid DNA, detected as a subG1 peak via flow cytometry.

#### ***Translocation of AIF***

**Figure 4:**
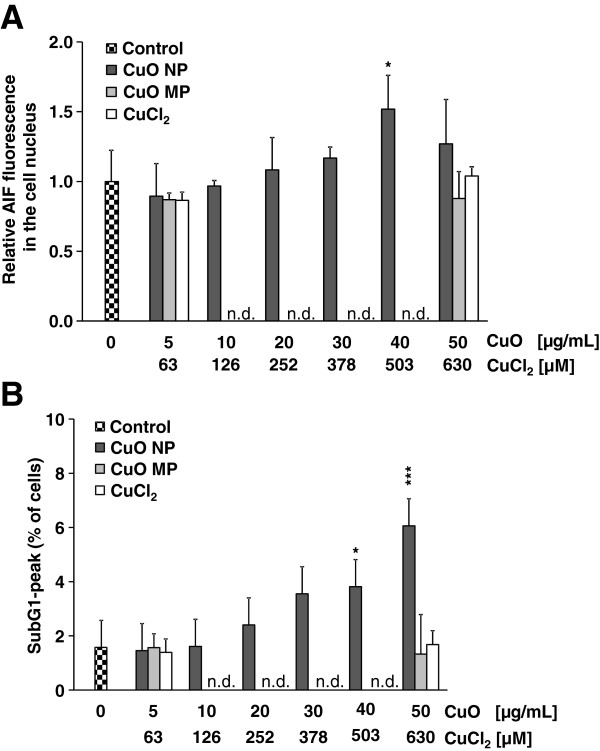
**Extent of apoptosis determined by subdiploid DNA and nuclear localization of AIF.** Logarithmically growing A549 cells were seeded, incubated with CuO NP, CuO MP or CuCl_2_ for 24 h, fixed and **(A)** stained with antibodies against AIF for fluorescent microscopy (evaluation of at least 50 cells per condition) or **(B)** stained with DAPI for flow cytometry analysis of sub-G1 peak (evaluation of 10 000 cells per condition) as described in Materials and methods. The data represent mean values of at least 3 independent experiments + SD. Statistically significant different from control: *p < 0.05, **p < 0.01, ***p < 0.001 as determined by unifactorial analysis of variance (ANOVA) and Dunnett’s T3 test. 1 μg/mL are equal to 0.2 μg/cm^2^ and 50 μg/mL CuO are equal to 630 μM Cu^2+^ in case of complete dissolution. n.d.: not determined.

To investigate the intracellular localization of AIF in A549 cells after exposure to CuO NP, CuO MP or CuCl_2_, fluorescence-labelled antibodies were applied and the cellular location of AIF was determined by fluorescence microscopy. After 8 h, 16 h or 24 h incubation with CuO NP, a slight concentration-dependent increase of AIF translocation in the cell nucleus was observed, resulting in 1.29, 1.33 and 1.52 fold fluorescence values (40 μg/mL CuO NP) over the control, respectively. In contrast, CuO MP and CuCl_2_ caused no AIF translocation into the cell nucleus at any time point investigated. The results after 24 h are presented in Figure 4A. The positive control staurosporine enhanced the control fluorescence to the 2.21, 2.33 and 2.17 fold values after 8, 16 or 24 h, respectively.

#### ***Impact on caspase 3/7 activity***

An alteration in the activity of the effector caspases 3 or 7 after 24 h incubation with CuO NP, CuO MP or CuCl_2_ was analysed by applying a luciferase-based assay as described in Materials and methods. None of the copper compounds affected caspase 3 or caspase 7 activities in A549 cells (data not shown). The positive control staurosporine increased the caspase activities 8.7-fold.

#### ***Accumulation of sub-diploid DNA (subG1 peak)***

As a third parameter of late apoptosis, the appearance of a subG1 peak was investigated at different time points by flow cytometry. While neither CuO MP nor CuCl_2_ increased the control value of less than 2%, CuO NP provoked a dose-dependent increase up to 6.1% cells containing subdiploid DNA at 50 μg/mL CuO NP after 24 h (Figure 4B). The appearance of the enlarged subG1 peak was also time-dependent, starting at 8 h, increasing at 16 h (data not shown) and being most pronounced after 24 h. The positive control staurosporine induced subdiploid DNA in 11.4% of cells after 24 h.

### Direct and indirect genotoxicity

To determine and compare the genotoxicity of the three copper compounds, four parameters have been included. First, the potency to induce DNA strand breaks has been determined by Alkaline Unwinding. Second, since copper may catalyse Fenton-type reactions, the induction of DNA strand breaks under pro-oxidative conditions, i.e., elevated levels of H_2_O_2_, was investigated. Third, the induction of micronuclei was determined as a measure of chromosomal damage. Finally, the impact on poly(ADP-ribosyl)ation was explored, since this reaction has been shown previously to be inhibited at comparatively low concentrations of water soluble copper [6], indicative of indirect genotoxicity via inhibition of enzymes involved in maintaining genomic stability.

#### ***Induction of DNA strand breaks***

**Figure 5:**
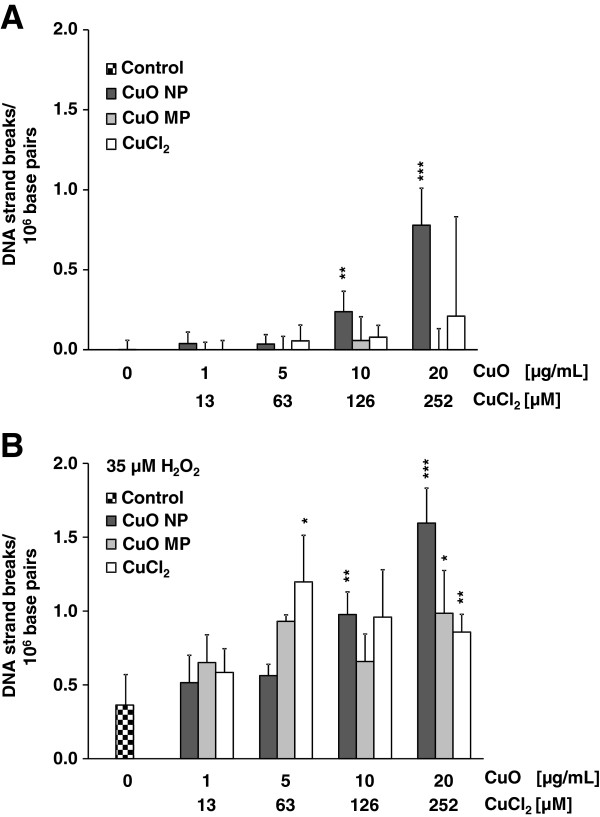
**Induction of DNA strand breaks by CuO NP, CuO MP or CuCl**_**2 **_**in the absence and presence of H**_**2**_**O**_**2**_**.** Logarithmically growing HeLa S3 cells were preincubated with CuO NP, CuO MP or CuCl_2_ for 24 h alone **(A)** or in combination with 35 μM H_2_O_2_**(B)** for 5 minutes in the continued presence of CuO NP, CuO MP or CuCl_2_. DNA strand breaks were quantified by Alkaline Unwinding as described in Materials and methods. The data represent mean values of 3 determinations + SD. Statistically significant different from control **(A)** or from H_2_O_2_-treated cells in the absence of copper compounds **(B)**: *p < 0.05, **p < 0.01, ***p < 0.001 as determined by unifactorial analyis of variance (ANOVA) followed by Dunnet's T3 test. 1 μg/mL are equal to 0.2 μg/cm^2^ and 20 μg/mL CuO are equal to 252 μM Cu^2+^ in case of complete dissolution.

After 24 h incubation CuO NP induced DNA strand breaks in HeLa S3 cells in a concentration-dependent manner, starting at the non-cytotoxic concentration of 10 μg/mL and reaching 0.8 DNA strand breaks/10^6^ base pairs at 20 μg/mL. No significant induction was seen in case of CuO MP or with CuCl_2_ (Figure 5A). However, in the presence of elevated levels of H_2_O_2_ all three copper compounds increased the number of DNA strand breaks. While treatment with 35 μM H_2_O_2_ for 5 min alone revealed 0.4 DNA strand breaks/10^6^ base pairs, this value was increased most pronounced by CuO NP up to 1.6 DNA strand breaks/10^6^ base pairs. Under these pro-oxidative conditions, also CuO MP and CuCl_2_ caused significant elevations of H_2_O_2_-induced DNA strand breaks (Figure 5B).

#### ***Induction of micronuclei***

**Figure 6:**
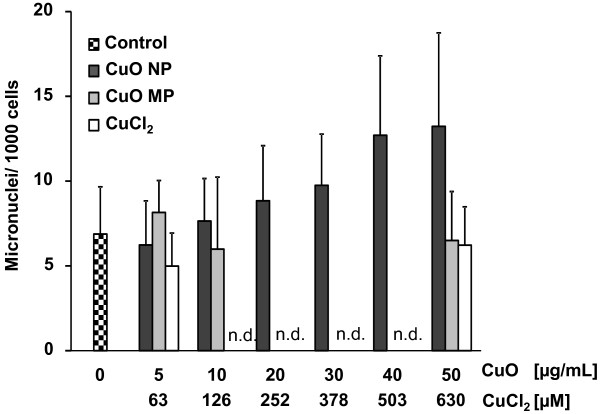
**Induction of micronuclei in A549 cells by CuO NP, CuO MP or CuCl**_**2**_**.** Logarithmically growing A549 cells were incubated with CuO NP, CuO MP or CuCl_2_ for 24 h. The frequencies of micronuclei were determined according to [26] as described in Materials and methods. The data represent mean values of at least 3 independent experiments + SD. 1 μg/mL are equal to 0.2 μg/cm^2^ and 50 μg/mL CuO are equal to 630 μM Cu^2+^ in case of complete dissolution. n.d.: not determined.

The formation of micronuclei (MN) was determined by a flow cytometric approach established by Bryce et al. [26]. The evaluation of micronuclei is based on a two-colour-fluorescence staining of the DNA to discriminate between micronuclei and DNA fragments generated during apoptosis or necrosis. In a first step, the red dye ethidium bromide monoazide (EMA) penetrates necrotic and apoptotic cells due to their damaged cell membrane and binds to nucleic acids. Subsequently, the cells are lysed and their nucleic acids are stained with SYTOX Green. Consequently, the necrotic and apoptotic cells exhibit a double staining and due to different wavelengths of emission it is possible to discriminate between micronuclei (green) and DNA fragments from apoptotic or necrotic cells (red/ green). The positive control 10 J/m^2^ UVC induced 105 micronuclei/1000 cells. Even though there was a tendency towards higher frequencies of micronuclei in case of cytotoxic concentrations of CuO NP, this effect was not significant and would not be informative at these levels of toxicity; neither CuO MP nor CuCl_2_ induced micronuclei at concentrations up to 50 μg/mL (CuO) or 630 μM (CuCl_2_) (Figure 6).

#### ***Effect on poly(ADP-ribosyl)ation***

**Figure 7:**
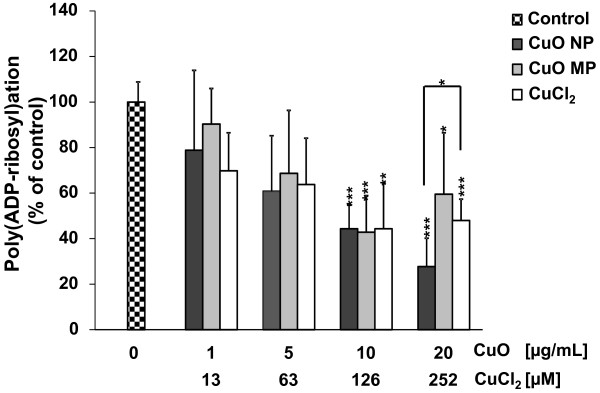
**Inhibition of H**_**2**_**O**_**2**_**-induced poly(ADP-ribosyl)ation by CuO NP, CuO MP or CuCl**_**2**_**.** Logarithmically growing HeLa S3 cells were preincubated with the respective copper compound for 24 h and treated with 100 μM H_2_O_2_ for 5 minutes in the continued presence of CuO NP, CuO MP or CuCl_2_. Afterwards, cells were washed, fixed and processed as described in Materials and methods. The data represent mean values of at least 6 determinations based on the evaluation of at least 50 cells + 95% confidence intervall (CI). Statistically significant different from control: *p < 0.05, **p < 0.01, ***p < 0.001 as determined by unifactorial analyis of variance (ANOVA) followed by Dunnett T/T3 test in case of homogeneous (CuO NP, CuCl_2_)/ inhomogeneous (CuO MP) variances. Significant difference between 252 μM CuCl_2_ and 20 μg/ml CuO NP as determined by Students’ *t*-test: *p < 0.05. 1 μg/mL are equal to 0.2 μg/cm^2^ and 20 μg/mL CuO are equal to 252 μM Cu^2+^ in case of complete dissolution.

To analyse the extent of poly(ADP-ribosyl)ation we determined the formation of poly(ADP-ribose) after activation with H_2_O_2_ immunologically, by applying the highly specific monoclonal antibody 10H against poly(ADP-ribose) and a secondary FITC-conjugated antibody. CuO NP, CuO MP as well as CuCl_2_ decreased the extent of H_2_O_2_-induced poly(ADP-ribosyl)ation concentration-dependent to around 44% at non-cytotoxic concentrations of 10 μg/mL CuO or 126 μM CuCl_2_. While no further inhibition was observed in case of CuO MP and CuCl_2_, incubation with 20 μg/mL CuO NP inhibited poly(ADP-ribosyl)ation most pronounced to a residual activity of 28% (Figure 7).

### Cellular uptake and intracellular bioavailability

**Figure 8:**
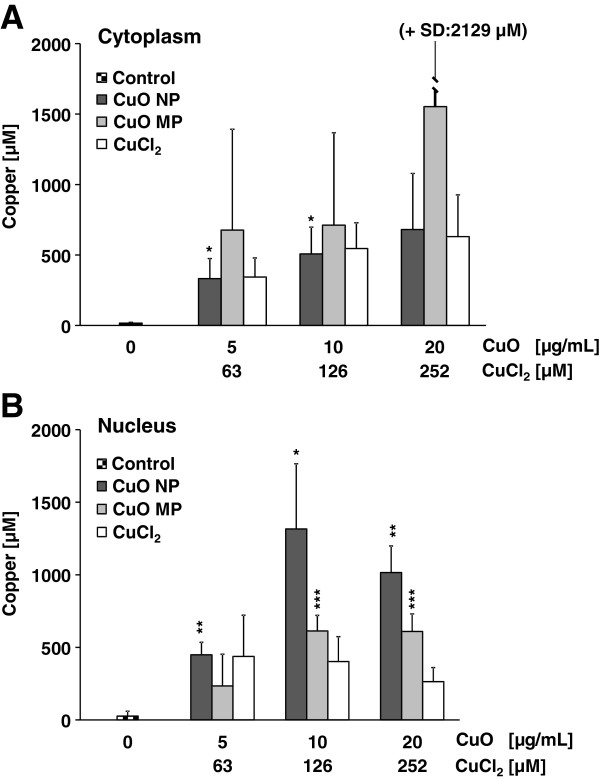
**Copper bioavailability and intracellular distribution after incubation with CuO NP, CuO MP or CuCl**_**2 **_**in A549 cells.** Logarithmically growing A549 cells were incubated for 24 h with CuO NP, CuO MP or CuCl_2_. Subsequently, **(A)** nuclear and **(B)** cytoplasmic protein fractions were isolated and the copper content was quantified by ICP-MS as described in Materials and methods. The data represent mean values of at least 3 independent experiments + SD. Statistically significant different from control: *p < 0.05, **p < 0.01 as determined by unifactorial analysis of variance (ANOVA) and Dunnett’s T3 test. 1 μg/mL are equal to 0.2 μg/cm^2^ and 20 μg/mL CuO are equal to 252 μM Cu^2+^ in case of complete dissolution.

One important aspect which may relate to the observed differences in cyto- and genotoxicity of CuO NP as opposed to CuO MP are potential differences in uptake, intracellular distribution and intracellular deliberation of copper ions, which may in turn provoke copper overload in cells. Since it is difficult to remove all particles from extra- and intracellular membranes to prevent artifacts in copper ion quantification, within the present study an approach was chosen where only copper present in the soluble fractions of the cytoplasm and the nucleus were determined. Thus, to quantify the bioavailability and intracellular distribution of ionic copper derived from dissolution of CuO particles as well as from CuCl_2_, A549 cells were incubated with the respective copper compound for 24 h. Subsequently, the cells were lysed and the soluble cytoplasmic fraction was isolated, followed by lysis of the nuclei to derive the soluble nuclear fraction. Copper contents were calculated based on cell or nuclei volume as well as on the protein content of the respective fraction. The basal copper level of A549 cells was found to be 15 μM in the cytoplasmic and 27 μM in the nuclear fraction. Treatment with either CuO NP or CuCl_2_ provoked a concentration-dependent copper accumulation in the cytoplasmic fraction. Thus, the lowest incubation concentration of 5 μg/mL CuO NP or 63 μM CuCl_2_ increased the basal cytoplasmic copper level by 22-fold to around 330 μM, reaching 630 μM at 252 μM CuCl_2_ and 680 μM at 20 μg/mL CuO NP. In case of CuO MP the cytoplasmic copper content was quite variable and lead to very high standard deviations. In the nuclear fraction, highest copper concentrations of more than 1 mM were reached after treatment with 10 or 20 μg/mL CuO NP, while CuO MP yielded around 600 μM at the same incubation concentrations. Lowest levels were observed after treatment with CuCl_2_, reaching about 400 μM, but lacking a clear dose-dependency (Figure 8A,B).

## Discussion

The results presented in this study confirm previous observations with respect to the distinct cytotoxicity of CuO NP; aim of the present study was to elucidate the underlying mechanism(s). Reasons for the particularly high cytotoxicity of CuO NP may be a direct interaction of undissolved particles with cellular components including the plasma membrane, perhaps facilitated by the 23-fold higher surface area of the CuO NP when compared to the CuO MP, or by copper ions deliberated either extracellularly or intracellularly. Even though copper is an essential trace element, elevated intracellular levels may exceed copper homeostasis, giving rise to pro-oxidative reactions. Thus, within this study, three different copper compounds, namely CuO NP, CuO MP and water soluble CuCl_2_, were, based on their total copper content, systematically compared with respect to cytotoxicity, their dissolution in extra- and intracellular media, the actual intracellular and intranuclear concentrations reached upon treatment of A549 cells as well as oxidative stress-mediated genotoxicity. The data demonstrate that in spite of pronounced differences in cytotoxicity all copper compounds investigated are in principle bioavailable; as for water soluble copper, this leads to highly elevated intracellular copper levels also in case of the particulate compounds. Among the three copper compounds, CuO NP revealed higher concentrations in the nucleus. While the differences in intracellular bioavailability are not sufficient to explain the differences in cytotoxicity, especially the copper accumulation in the nucleus appears to correlate with the extent of genotoxicity.

To assess the cytotoxicity, the colony forming ability was investigated as a sensitive parameter of long-term toxicity in A549 and HeLa S3 cells. While no toxicity was observed in case of CuO MP in the concentration range applied up to 50 μg/mL, pronounced dose-dependent toxicity was observed after 24 h incubation with CuO NP or CuCl_2_ in both cell lines. While CuO NP exerted similar effects in A549 and HeLa S3 cells, CuCl_2_ was slightly less toxic in A549 cells. In principle, with respect to CuO NP and CuO MP, the results confirm previous observations on the cytotoxicity of differently sized CuO in mammalian cells [20,21,27]. Nevertheless, they contradict in part observations by Karlsson et al. [19] where equimolar levels of CuCl_2_ were eight times less cytotoxic to A549 cells than CuO NP after 18 h incubation. However, these authors applied trypan blue exclusion as a measure of cytotoxicity, which may be less sensitive when compared to colony forming ability applied in the present study. Concerning the mechanism of cell death, pronounced differences were seen between CuO NP and CuCl_2_: Only CuO NP induced significant elevations of the SubG1-peak, indicative of apoptosis, while no effect was seen in case of CuCl_2_. Furthermore, CuO NP caused a slight increase in AIF nuclear translocation, pointing towards mitochondrial membrane damage. These observations agree with previous studies on the translocation of phosphatidylserine by CuO NP [16] and the depolarization of the mitochondrial membrane potential by CuO NP [17,20]. Mitochondrial damage may be the consequence of direct interactions with undissolved particles after endocytotic uptake and/or by ROS-derived lipid peroxidation resulting in disturbed membrane integrity and the release of apoptotic enzymes.

To elucidate the impact of released copper ions on the cytotoxicity, the dissolution of CuO NP and CuO MP in different model fluids was quantified. Decisive parameters both for the dissolution and for agglomeration/aggregation may be the composition of buffers and cell culture media, the presence of proteins, for example due to the addition of fetal calf serum, as well as the pH. We found a higher solubility in case of CuO NP when compared to CuO MP in bidistilled water and in PBS; however both copper oxides dissolved only sparsely (< 3%), in agreement with two previous studies [21,28] and the reported hydrophobicity of CuO NP [29]. Since the lung is one important target organ for CuO particle toxicity, also artificial alveolar fluid (AAF) was included; again, the solubility was higher for CuO NP than for CuO MP, but was altogether very low with values below or around 1%. Thus, CuO NP and CuO MP dissolved only marginally in aqueous liquids at neutral pH. With respect to cell culture experiments we investigated the dissolution of CuO NP and CuO MP in DMEM and DMEM/FCS. Here, the dissolution was much higher for both particle types. In DMEM/FCS, CuO NP dissolved ten times stronger (44%) than CuO MP (4%) after 24 h in good agreement with two former studies [16,30]. For comparison, in DMEM without FCS dissolution was even higher (66% in case of CuO NP and 27% in case of CuO MP). Concerning DMEM, the accelerated dissolution may be explained by the presence of copper ion complexing amino acids; the addition of fetal calf serum may result in a protein corona around the particles [12,31], which in this case appears to protect both types of particles from dissolution. However, one has also to consider the conditions in different intracellular compartments. Both nano- and microsized particles have been shown to be taken up via endocytosis, involving the formation of endosomes (pH 6.2), and their transformation into lysosomes (pH 4.5) [15]. Thus, within this study, the dissolution was followed in the acidic environment of an artificial lysosomal fluid (ALF) for 30 minutes to 7 d. CuO NP dissolved almost completely after 2 h in ALF at pH 4.5; nevertheless, it took 7 d to dissolve CuO MP. With respect to the former particles, dissolution in ALF was even faster when compared to 3 d required for dissolution of CuO NP in a complexing bis-Tris solution reported in literature [28]. The data demonstrate the relevance of comprehensive model studies related to intra- and extracellular dissolution of particles; an estimation based on the largely insolubility of CuO particles in bidistilled water or buffer would not allow to detect the pronounced differences between nano- and microscale particles.

With respect to the intracellular bioavailability, two aspects have to be considered, namely the internalization of particles and the increase in water soluble copper ions within the cell. Concerning the uptake of particles, three studies investigated the uptake of CuO NP into human cells by TEM [16-18]. Thus, Wang et al. observed aggregates of CuO NP in lysosomes, mitochondria and the cell nucleus and, by applying an endocytosis inhibitor, demonstrated the importance of endocytosis for particle uptake [17]. Within the present study we aimed to elucidate the impact of the three copper compounds on the levels of copper ions in the cytoplasm and the cell nucleus. Since copper particles associated with plasma and intracellular membranes are difficult to remove and would potentially lead to artifacts with respect to ionic copper concentrations, we applied a protocol where successively cytoplasmic and nuclear soluble fractions were isolated [32]. The principle consists in lysis of the plasma membrane and subsequent centrifugation; the supernatant contains the soluble cytoplasmic fraction. Subsequently, the precipitated nuclei are lysed and centrifuged to obtain the soluble nuclear fraction in the supernatant. For all three copper compounds, we observed a pronounced increase in copper concentrations both in the cytoplasm and the nucleus of A549 cells. Concerning CuO NP and water soluble copper chloride, the basal copper concentration in the cytoplasm was accelerated up to 45- and 42-fold and about 38- and 15-fold in the nucleus, respectively. In case of CuO MP the standard deviation between experiments was very high, which may be due to the presence of incompletely dissolved particles in the lysosomes; here, small differences in particle numbers would give rise to extended variations in the copper content. In support of this theory, error bars in case of CuO NP are much smaller, which could be explained by their much faster dissolution in the lysosomes. With respect to the nuclear fraction, highest concentrations were obtained in case of CuO NP, reaching 1.3 mM copper. These findings appear to contradict results by Wang et al. [17] as well as by Cronholm et al. [18] who reported about 20-fold (Wang) or 40 fold (Cronholm) higher intracellular concentrations in case of CuO NP. The pronounced differences may be due to the applied preparation techniques. As indicated above, in the present study the soluble fractions of both cellular compartments were isolated, while Wang and coworkers as well as Cronholm and coworkers used washing and centrifugation procedures before GF-AAS measurements of metal content; by this approach, particles may not have been completely removed from the plasma membrane or intracellular membranes. Nevertheless, Wang et al. also found unusual high values for copper in control cells, while basal copper levels of around 20 μM observed in the present study are in the range of values reported previously for mouse hepatocytes [33].

One key mechanism of copper toxicity consists in its redox activity, leading to Fenton-type reactions in the presence of H_2_O_2_ and generating highly reactive hydroxyl radicals. Damage to cellular macromolecules including DNA can occur if the storage capability for copper is exceeded, e.g. due to overload conditions, or if oxidative stress induces a sudden release of copper from metallothionein [1,34]. In the present study, the induction of DNA strand breaks was investigated as an indicator of oxidative stress. CuO NP induced DNA strand breaks in HeLa S3 cells already at non-cytotoxic concentrations of 10 μg/mL, whereas the induction by CuCl_2_ and CuO MP was negligible. With respect to CuO NP, our results are consistent with former studies where the authors detected elevated levels of DNA damage by CuO NP using the comet assay [17,19,21,24] and which may in addition to the highly enhanced intracellular copper levels also be due to increased mitochondrial damage [17,20], facilitating Fenton-type reactions. Regarding water soluble CuCl_2_, our data seem to contradict previous results where water soluble CuSO_4_ induced DNA strand breaks [6]; nevertheless, this effect was restricted to cytotoxic concentrations > 300 μM, while the highest concentration included in our study was around 250 μM and still in the non-cytotoxic concentration range. Interestingly, all three compounds under investigation caused an increase in the number of H_2_O_2_-induced DNA strand breaks, indicative of an augmentation of pro-oxidative conditions within the cell. With regard to DNA damage on the chromosomal level, only CuO NP exerted a tendency of increased micronuclei formation. At the highest concentration investigated the number of micronuclei was non-significantly doubled. With respect to CuO NP our results do not resemble those observed in vivo [22] and also appear to contradict significantly enhanced levels of micronuclei in a neuroblastoma cell line and RAW 264.7 cells [23,24]. Nevertheless, the latter may be explained by different experimental approaches. Thus, Perrault and coworkers [23] as well as Di Bucchianico and coworkers [24] collected cells by cytochalasin B, and thereby detected micronuclei which arise either by clastogenic or aneugenic effects. In contrast, the flow cytometric approach conducted within the present study is restricted to the detection of clastogenic effects.

One further aspect investigated within the present study is the impact of copper-based particles on poly(ADP-ribosyl)ation. Poly(ADP-ribosyl)ation is one of the earliest events following the induction of DNA strand breaks and is catalysed by members of the poly(ADP-ribose)polymerase (PARP) super family, mostly by PARP-1 [35]. Poly(ADP-ribosyl)ation is a posttranslational modification of proteins, where multiple ADP-ribose moieties derived from NAD^+^ are covalently attached to acceptor proteins like histones, transcription factors or DNA repair proteins. This reaction is thought to mediate DNA damage signalling and plays an important role in maintaining genomic stability, best understood for base excision repair (BER) and the initial steps of single strand break repair (SSBR) [36]. PARP-1 contains three zinc-binding domains, two of which are required for DNA binding [37], thereby protecting DNA strand breaks from conversion into more disadvantageous lesions, e.g. via attack of nucleases and/or recombination [38,39]. Previous studies in our laboratory identified PARP-1 as well as poly(ADP-ribosyl)ation as sensitive target of water soluble copper sulfate [6]; thus, within the present study this endpoint was included to assess the impact of particulate copper compounds. The results demonstrate a dose-dependent inhibition by all three copper compounds, starting at non-cytotoxic concentrations; nevertheless, the strongest inhibition to about 28% residual activity was seen in case of CuO NP. In principle, different mechanisms could apply for the observed inhibition of poly(ADP-ribosyl)ation. As PARP-1 is activated upon the recognition and binding to DNA strand breaks, an inhibition could be due to a diminished induction of DNA strand breaks in combination with H_2_O_2_. Nevertheless, this can be excluded since all three copper compounds even increased the frequency of H_2_O_2_-induced DNA strand breaks under the same incubation conditions. Another reason could be a depletion of NAD^+^, the substrate of PARP-1. Nevertheless, since the inhibition was observed at non-cytotoxic concentrations where neither cell growth nor cell division was impaired, this reason seems unlikely as well. Also, CuO MP did not affect the cell growth of HeLa S3 cells at any concentration and yet was still inhibitory towards poly(ADP-ribosyl)ation. The most likely explanation is a direct inhibition of PARP-1 by copper ions under overload conditions. Thus, in a previous study we observed a diminished activity of isolated PARP-1 by copper sulfate [6]. Copper ions are redox-active and may react with redox-sensitive amino acids. Potential targets may be thiol groups in cysteines, for example those involved in zinc complexation within the zinc binding structures of PARP-1, leading to zinc deliberation and unfolding of the respective domains required for the recognition of DNA strand breaks and the catalytic activity of PARP-1 [37,40]. Also, copper ions may bind directly to thiols, thus leading to structural alterations. The more pronounced inhibition by CuO NP as compared to CuO MP and CuCl_2_ may be explained by elevated copper levels in the nucleus observed in case of CuO NP.

## Conclusion

In summary, the results presented in this study support the high cytotoxicity of CuO NP as described previously. By systematic comparison of CuO NP, CuO MP and water soluble copper chloride, the impact of particle size, extracellular solubility and intracellular dissolution and thus bioavailability of copper ions on cytotoxicity as well as direct and indirect genotoxicity was investigated. With regard to cytotoxicity, the total copper content and the intracellular copper levels appear to be of minor importance, since CuO MP are not cytotoxic and yet increase nuclear copper levels to a higher extent than CuCl_2_, which is clearly toxic based on the same copper content. CuO NP were most cytotoxic and the only compound inducing apoptosis. Thus, the high cytotoxicity is most likely related to particle characteristics like the high surface area (about 23-fold higher when compared to CuO MP), which may facilitate redox reactions either intra- or extracellularly, leading to cell death. Additionally, it has to be taken into account that a considerable fraction of the nanoparticles but not of the microparticles has been dissolved already in the incubation medium; therefore copper ions either present or released extracellularly may contribute to the cytotoxicity as well. Concerning genotoxicity, especially the copper levels in the nucleus appear to be relevant. Here, highest levels were observed in case of CuO NP, and this was also the only compound which generated DNA strand breaks in the absence of (additional) H_2_O_2_ and exerted the most pronounced inhibition of poly(ADP-ribosyl)ation. Taken together, different features of CuO NP appear to affect cyto- and genotoxicity, and especially the intra-nuclear bioavailability of copper ions exceeding cellular copper homeostasis may impair genomic stability.

## Materials and methods

### Particles and metal compounds

CuO NP (#544868, Lot #MKAA0633), CuO MP (#208841, Lot # MKAA1788) and CuCl_2_ (#307483) were purchased from Sigma-Aldrich Chemie GmbH (Steinheim, Germany). Storage occurred in containers of amber glass (CuO NP) or High Density Polyethylene (HD-PE) (CuO MP) in dry places at room temperature (RT). The particulate materials were characterized using DLS with respect to size, scanning electron microscopy (SEM) for size and morphology, BET for surface area, ZP for surface charge, ICP-MS, EDX and oxygen analysis for purity and composition as well as X-ray Diffraction (XRD) for crystallinity. The particles were also investigated with respect to their impact on pH in relevant media. Finally, an endotoxin contamination was excluded.

### Metrics

The particle dose is stated in mass concentration [μg/mL]. For the purpose of comparison the conversion into other common metrics as area-related mass [μg/cm^2^], surface area concentration [cm^2^/mL] and molar concentration [μM] is given in Table 1.

**Table 1:** Metrics

**CuO**	**CuO**	**Copper**	**CuO NP**	**CuO MP**
**[μg/mL]**	**[μg/cm**^**2**^**]**	**[μM]**	**[cm**^**2**^/mL]*	**[cm**^**2**^/mL]*
1	0.2	13	0.1723	0.0074
5	1	63	0.8612	0.0369
10	2	126	1.723	0.0738
20	4	252	3.446	0.1476
30	6	377	5.169	0.2214
40	8	503	6.892	0.2952
50	10	630	8.615	0.3690

Conversion of the applied doses from μg particles/mL suspension media (μg/mL) into μg particles/cm^2^ growth area of a cell culture dish (μg/cm^2^) into μmol copper/L suspension media (μM) and cm^2^ particle surface/mL suspension media (cm^2^/mL).

*Calculated from the specific surface area measured by BET analysis according to [41].

### Preparation of endotoxin-free materials

Snap-on lid glasses equipped with adequate teflon jacketed stirring bars were utilized for preparing the particle incubation suspensions. Prior to use, the glasses and stirring bars were rinsed with sterile filtered ultrapure water (H_2_O) (18 mΩ) to remove inorganic contaminations, followed by treatment with 70% ethanol prepared with sterile filtered H_2_O. Endotoxin contamination was excluded by dry sterilization for either 0.5 h at 250°C or 5 h at 220°C. The lids were cleaned as stated above and stored in 70% ethanol; prior to use they were dried in a sterile laminar airflow.

### Particle incubation suspensions

For all experiments incubation suspensions of particles were prepared following a standard operating procedure (SOP) published by the German Nano Care consortium [42]. Particles, received as dry powder, were aliquoted by weighing (scale: BP 61 S, Sartorius, Göttingen, Germany) into colorless, endotoxin-free 1.5 mL polystyrene reaction tubes (#72.690.001 Sarstedt, Nümbrecht, Germany). Stock solutions of 0.5-20 mg/mL CuO were prepared by transferring an aliquot completely into an endotoxin-free snap-on lid glass containing a stirring bar and replenishing with bidistilled water or medium with or without serum depending on the requirements of the respective experiments to the designated concentration. Each particulate compound and the control media were prepared by applying separate sets of stirring bars. Stock solutions were stirred on a multiphase stirrer (Variomag® Poly, Carl Roth GmbH, Karlsruhe, Germany) for 1 h and 900 rpm at RT. Dilutions in the range of 1–50 μg/mL (cell culture experiments), 100–500 μg/mL (endotoxin tests) and 50 – 6000 μg/mL (DLS/ZP) were prepared by adding aliquots of the stirring stock solution into snap-on lid glasses filled with adequate volumes of fresh medium. Stirring occurred for 24 h at 900 rpm and room temperature prior to use. CuCl_2_ was dissolved in bidestilled H_2_O (0.4 M) and sterile filtered (0.22 μm, cellulose acetate). Adequate dilutions were prepared directly before incubation. CuCl_2_ incubation solutions were prepared as stated above by stirring adequate stock solutions and dilutions at 900 rpm before application.

### Cell culture reagents

DMEM, trypsin and penicillin-streptomycin solutions are products of Sigma-Aldrich. FCS (#10270, Lot #41Q7361K) is a product of Invitrogen GmbH (Darmstadt, Germany). Leupeptine, phenylmethanesulfonyl-fluoride (PMSF), all salts, acids and bases, snap-on lid glasses and stirring bars were obtained from Carl Roth GmbH. Biochrom AG (Berlin, Germany) delivered cell culture dishes and flasks.

### Cell lines and cell culture

The adherent human cancer cell lines A549 (human lung adenocarcinoma cell line) and HeLa S3 (human cervix carcinoma cell line), both derived from ATCC, were maintained and grown as monolayer in DMEM supplemented with 10% FCS, containing 100 Units/mL penicillin and 100 μg/mL streptomycin (DMEM/FCS). Incubation took place in an atmosphere of 5% CO_2_ in air at 37°C and 100% humidity (HeraSafe, Thermo Scientific, Langenselbold, Germany). For all experiments cells were seeded at a density of 16,600 cells/cm^2^. After one day the supernatant from the logarithmically growing cells was removed and replaced by the particle incubation suspensions (0.2 mL/cm^2^) as indicated for the respective experiments.

### DLS and ZP

To determine the hydrodynamic particle size distribution by DLS and the ZP at 20°C, a Malvern Zetasizer Nano ZS (Malvern, Herrenberg, Germany), equipped with a 532 nM laser, was applied. 1.5 mL of the respective particle suspension was transferred into a clean square polystyrene cuvette (#67–754, Sarstedt). In two independent experiments, concentrations were optimized to the devices’ performance needs and ten replicates of at least two dilutions per particle were quantified. Measurement conditions such as distance from cuvette wall, number of runs and measurement duration were optimized for each particle. The implemented Zetasizer Nano ZS Dispersion Technology Software (DTS) Version 6.20 evaluated the data as intensity (iPSD), volume (vPSD) and number distribution (nPSD) in combination with parameters like the polydispersity index (PdI) ranging from 0 – 1. Thus, 0 reflects a monodisperse and 1 a polydisperse sample. A prerequisite to obtain the best possible result in terms of iPSD, vPSD and nPSD, is the knowledge of the physical characteristics of medium and particles. In the context of this work the refractive index (n), the viscosity (η), the dielectric constant (ϵ) and the density (ρ) of the cell culture media were obtained as described below for DMEM and DMEM/FCS. DMEM: n = 1.33, η [mPas] = 1.09, ρ [g/cm^3^] = 1.00, ϵ = 77.9548; DMEM/FCS: n = 1.33, η [mPas] = 1.14, ρ [g/cm^3^] = 1.00, ϵ = 77.5896; the refractive index of CuO is 2.58 [43]. Absorption of the CuO particles was approximated with 0.9 as proposed by Malvern for black particles.

ZP measurements were performed in the folded capillary cell DTS1061C (Malvern Instruments Ltd, Worcestershire, UK), rinsed with H_2_O and ethanol before adding the particle suspensions. For calculation of the ZP from the initially acquired electrophoretic mobility, the Smoluchowski approximation for polar solvents was used. In two independent experiments 3 replicates of at least two dilutions were analysed.

### Viscosity

Using the rheometer Physica MCR 301 (Anton Paar, Graz, Austria) the viscosity was determined at 20°C. The measurement program contained the following steps: 33 measurements of 5 s each at γ = 0.1…100 1/s, 10 measurements of 5 s each at γ = 100 1/s and 33 measurements of 5 s each at γ = 100…0.1 1/s. Calculation of viscosity was conducted with the software RHEOPLUS/32 Multi3 V 3.4 applying the evaluation method Newton I.

### Density

Density was evaluated using a pycnometric procedure, containing the following steps: equilibrating the empty or filled pycnometer to 20°C for 30 min, drying and weighing (scale: VWR 1502, Sartorius, Göttingen, Germany). After repeating the procedure with H_2_O and DMEM subsequent calculation resulted in the relative density in g/cm^3^.

### Refractive index

The refractive indices of DMEM and DMEM/FCS were determined at 20°C using a standard refractometer (Carl Zeiss AG, Oberkochen, Germany).

### Dielectric constant

The dielectric constants of DMEM and DMEM/FCS were determined using the 85070E Dielectric Probe Kit (Agilent Technologies, Inc., Santa Clara, CA, USA). Based on the molecular structure of the materials, the dielectric properties can be determined by a probe transmitting a signal in the range of radio frequency to microwave energy (200 MHz to 50 GHz) into the material under investigation. A network analyser calculates and displays the complex permittivity including the dielectric constant. Analysis was carried out after calibrating the device against a short circuit, air and H_2_O followed by immersion of the probe into the sample. The dielectric constant was given as a dimensionless number.

### Oxygen content

Oxygen content of the particles was determined using the N/O analyser TC 600 (LECO Instrumente GmbH, Mönchengladbach, Germany).

### TEM

Crystallinity was investigated by X-ray diffraction using a TECNAI G^2^ 20 S-TWIN (FEI, Hillboro, OR, USA) at an accelerating voltage of 200 kV. Briefly, an aliquot of the particle suspension in H_2_O was placed onto a carbon coated copper grid, dried and subsequently measured. Images were acquired and evaluated with DigitalMicrograph™ Software (Gatan, Inc., Pleasanton, CA, USA).

### SEM

Particle size, morphology and chemical composition of the powdered samples were investigated using a LEO 1530 Gemini (Carl Zeiss AG) in combination with EDX at an acceleration voltage of 10 kV. Before measurement, the particulate samples were suspended in H_2_O, applied onto a silicon specimen holder, dried and sputtered with platinum to increase conductivity.

### Specific surface area (BET)

Prior to measurements the powdered samples were dried for 6 d at 40°C in a vacuum drier (Binder GmbH, Tuttlingen, Germany). Analysis according to the BET [44] theory was conducted in a Gemini 2360 (Micromeritics GmbH, Aachen, Germany). Multipoint BET evaluation resulted in the specific surface area in m^2^/g.

### pH measurements

Particle suspensions were prepared as described above in H_2_O and DMEM/FCS. pH value was determined using the pH 330 pH-meter from WTW GmbH (Weilheim, Germany) equipped with a SenTix electrode.

### Endotoxin content

Particle suspensions with concentrations of 500 μg/mL were prepared in endotoxin-free water. The amount of endotoxin was measured by applying the ToxinSensorTM Endotoxin Detection System Kit (Genscript, Piscataway, USA) according to the manufacturers’ instructions.

### Solubility in model fluids

To access the copper ion release, CuO NP and CuO MP were immersed in H_2_O, DMEM, DMEM/FCS, PBS, AAF or ALF [45,46]. Stock suspensions in the respective media were prepared as described above. In case of DMEM and DMEM/FCS the stock suspensions were diluted to a final concentration of 50 μg/mL. Thereafter 10 mL were transferred into cell culture dishes and incubated for 2, 4, 8, 16 or 24 h at 37°C and 5% CO_2_. Subsequently, the suspensions were transferred into centrifuge tubes and centrifuged at 3000 × g followed by repeated centrifugation of the collected supernatants at 16000 × g. 1 mL of the resulting supernatant was concentrated by stepwise heating to 95°C to remove the water, decomposed by treatment with 1:1 HNO_3_ (69%)/H_2_O_2_ (31%) (v/v) (quality: suprapure), followed again by stepwise heating to 95°C. The crystalline residue was solubilized in 1 mL of H_2_O and analysed for copper content by GF-AAS (Pinaccle 900 T, Perkin Elmer, Rodgau, Germany). Potential adsorptive losses by this procedure were excluded by recovery experiments, yielding 103% copper in case of DMEM/FCS. Successful separation of particles from the liquid was verified by DLS (data not shown).

Solubility of CuO NP and CuO MP in H_2_O, PBS, AAF and ALF was investigated using a modified procedure established for the long-term incubation of up to 7 d. Particle stock suspensions in H_2_O, AAF and ALF were prepared as stated above, the respective dilutions (50 μg/mL) were prepared in 50 mL centrifuge tubes and agitated for 1, 4 or 7 d at 37°C using an incubation shaker (MKR 13, HLC-BioTech, Bovenden, Germany) at 100 rpm. Centrifugation and oxidative decomposition were carried out as stated above.

### Colony forming ability

Determination of colony forming ability provided details on acute toxicity in terms of cell number and long term toxicity. Logarithmically growing A549 or HeLa cells were incubated for the indicated times, trypsinized and collected in DMEM/FCS. After cell counting (Coulter Z2, Beckmann Coulter GmbH, Krefeld, Germany) triplicates of 300 cells per dish were seeded into fresh medium. After 7 d colonies were fixed, stained with Giemsa solution and counted.

### Subdiploid DNA

To measure the induction of apoptosis by means of the subG1 peak, cells were seeded, incubated with CuO NP, CuO MP, CuCl_2_ or as a positive control with 400 nM staurosporine for 4, 8, 16 or 24 h. Then the cells were trypsinized, collected in ice-cold PBS/5% FCS, combined with the supernatant and centrifuged (1500 rpm (448 × g), 5 min, 4°C). The pellet was resuspended in 1 mL cold PBS before 3 mL ice-cold ethanol were added under vortexing, followed by fixation overnight at -20°C. For flow cytometric analysis, the samples were centrifuged (1500 rpm, (448 × g), 5 min, RT), the pellet was resuspended in 1 mL DAPI dye solution (CyStainDNA, Partec) and incubated for 2 h at 4°C and 2 h at RT in the dark. 10 × 10^5^ cells per sample were analysed for the occurrence of a SubG1 peak using the software FloMax® (Partec).

### Activity of the effector caspases 3/7

The activity of the effector caspases 3 and 7 was measured using the Caspase-Glo® 3/7 Assay Kit (Promega Corporation, Madison, WI, USA). 5.5 × 10^3^ A549 cells were seeded into each well of a white flat-bottomed 96 well plate (Brand, Germany) and allowed to attach for 24 h before incubation with CuO NP, CuO MP, CuCl_2_ or 400 nM staurosporine as a positive control took place for another 24 h. Subsequently, the assay was conducted according to the instructions given by the manufacturer.

### AIF

Analysis of the AIF release and its translocation from the mitochondria to the cell nucleus was investigated by an immunofluorescent approach using a specific antibody against AIF in combination with a fluorescence-coupled secondary antibody. 12 mm coverslips were positioned into 40 mm cell culture dishes before 1.53 × 10^5^ A549 cells were seeded, allowed to attach for 24 h and incubated with CuO NP, CuO MP, CuCl_2_ or 400 nM staurosporine as a positive control, for 4, 8, 16 or 24 h. Subsequently, the culture dishes were positioned on ice, the incubation medium was removed, cover slips were washed three times with PBS (4°C) and fixed for 45 minutes in ice-cold 3.7% formaldehyde solution. Thereafter threefold washing with ice-cold PBS was followed by the addition of 0.25% Triton X-100 in PBS for 25 minutes and a further washing step. Unbound protein binding sites were blocked in PBS/5% FCS for 5 minutes at RT. The rabbit-polyclonal IgG antibody against AIF (Santa Cruz, Heidelberg, Germany) diluted in blocking buffer (dilution 1:50) was applied to the coverslips and incubated in a humid chamber (30 min, 37°C). Threefold washing in PBS and treatment for 10 minutes in blocking buffer at RT was followed by application of the secondary antibody (Goat-anti rabbit Cy3) in blocking buffer (dilution 1:300, 30 min, 37°C). Residuals were removed by threefold washing with PBS and coverslips were prepared on microscope slides by using VECTASHIELD Mounting Medium with DAPI (Biozol, Eching, Germany). The red fluorescence of AIF in the nucleus as well as the nuclei size were analysed and quantified using the Axio Imager.M1 (Zeiss, Oberkochen, Germany) and the software AxioVision version 4.8.

### Induction of DNA strand breaks

DNA strand breaks were quantified by Alkaline Unwinding as described previously [47]. Briefly, 1.53 × 10^5^ HeLa S3 cells were seeded in cell culture dishes (inner Ø 34 mm) and allowed to attach for 24 h. Subsequently, cells were incubated for 24 h with the respective substances alone as well as in combination with 35 μM H_2_O_2_ for 5 min. Afterwards, the medium was removed, cells were washed with ice-cold PBS, an alkaline solution (0.03 M NaOH, 0.02 M Na_2_HPO_4_, 0.9 M NaCl) was added and the DNA was allowed to unwind for 30 minutes in the dark. After neutralization and sonication, single- and double-stranded DNA were separated by performing hydroxyapatite chromatography at 60°C. Single-stranded DNA was eluted by 0.15 M and double-stranded DNA by 0.35 M potassium phosphate buffer. The addition of Hoechst 33258 at a final concentration of 7.5 × 10^-7^ M to each mL of sample and measurement of the fluorescence (excitation wave length 360 nm, emission wave length 455 nm) (Infinite M200 Pro, Tecan, Männedorf, Switzerland) was followed by quantification of DNA strand breaks as described previously [47].

### Flow cytometric scoring of micronuclei

Micronuclei were quantified via flow cytometry as described by Bryce et al. [26]. 31,000 A549 cells in 0.4 mL DMEM/FCS were seeded into each cavity of a 24 well plate and allowed to attach for 24 h. Subsequently, cells were incubated for 24 h with CuO NP, CuO MP or CuCl_2_. As a positive control, cells were irradiated with 10 J/m^2^ UVC (254 nm). After completion of postincubation (24 h) the plate was precooled on ice for 20 minutes before the medium was removed. Under exclusion of direct light, 300 μL ice-cold dye solution (8.5 μg/mL EMA (Invitrogen, Darmstadt, Germany)) in PBS/2% FCS) were added into each well. Irradiation of the plate without lid (30 min, on ice) with the light of a cold light halogen lamp (distance 15 cm) was followed by a washing step with 1 mL of cold buffer (PBS/2% FCS). Afterwards, 500 μL lysis solution A (58.4 mg/100 mL NaCl (10 M), 0.114 g/100 mL sodium citrate dihydrate (0.442 mM), 30 μL/100 mL IGEPAL, 0.5 mg/mL RNAse (Roche, Grenzach-Wyhlen, Germany), 0.4 μM SYTOX Green (Invitrogen)) was added and incubated in the dark (1 h, 37°C). Hereafter, 500 μL freshly prepared lysis solution B (8.56 g/100 mL sucrose (25 mM), 1.5 g/100 mL citric acid (7.81 mM), 0.4 μM Sytox Green) were added to each well and left for 30 minutes in the dark (RT). Finally the solution was resuspended by soft tapping, transferred into a measuring tube and applied to flow cytometric analysis on the Partec PAS (Partec, Görlitz, Germany). 30,000 cells per sample were analysed using the software FloMax®.

### Poly(ADP-ribosyl)ation

The impact on poly(ADP-ribosyl)ation was determined as described previously [48]. Briefly, HeLa S3 cells were grown as monolayers in cell culture dishes equipped with coverslips (18 mm diameter) for 24 h and subsequently incubated with the particle suspensions or CuCl_2_ for 24 h. Poly(ADP-ribosyl)ation was induced by treatment with 100 μM H_2_O_2_ for 5 minutes at 37°C. As blind values, untreated cells as well as cells treated with the respective copper compounds in the absence of H_2_O_2_ were included and fluorescence intensities were substracted from those of copper plus H_2_O_2_-treated cells. Afterwards, coverslips were removed from the cell culture dishes, washed in ice-cold PBS and fixed in ice-cold 10% (w/v) trichloroacetic acid for at least 15 min. Afterwards, successive 5 minutes washings in ice-cold 70%, 90% and 100% ethanol took place. Air-dried coverslips were rehydrated in PBS and incubated with the monoclonal antibody 10H directed against poly(ADP-ribose) in blocking buffer (5% skim milk powder in PBS) [49]. Incubation was carried out in a humid chamber at 37°C for 30 min, followed by fourfold washing of the coverslips in PBS. The secondary, FITC-conjugated anti-mouse antibody (Sigma-Aldrich) in blocking buffer (dilution 1:30) was applied accordingly. Finally, coverslips were mounted on glass slides in Vectashield mounting medium containing DAPI. Fluorescence intensity was evaluated using a Zeiss Axio Imager.Z equipped with the software AxioVision version 4.8. The software allowed the simultaneous determination of the colocalised nuclear DAPI staining and nuclear poly(ADP-ribose) fluorescence. At least 100 cells were selected for quantification of FITC fluorescence.

### Bioavailability and intracellular distribution

Soluble cytoplasmic and nuclear fractions of A549 cells were prepared using a method described previously [32]. Briefly, logarithmically growing A549 cells were incubated for the times indicated. The cells were trypsinized, collected in ice-cold PBS supplemented with 5% FCS, washed twice with PBS and counted electronically for cell number and cell volume. All following steps were carried out on ice. 2 × 10^6^ cells were allowed to swell in cell lysis buffer (0.01 M HEPES pH 7.9, 0.01 M KCl, 0.0015 M MgCl_2_, 0.3 M saccharose, 0.0005 M dithiothreitol (DTT), 0.0006 M phenylmethanesulfonfluoride (PMSF), 0.0065 mM Leupeptine) for 15 minutes before the addition of 25 μL 10% (v/v) IGEPAL CA-630 (#I3021, Fluka) in H_2_O for cell lysis. The mixture was vortexed for 10 s and the nuclei pelleted at 1500 × g (15 min, 4°C). The supernatant contained the soluble cytoplasmic fraction. The nuclei-containing pellet was washed twice with cell lysis buffer to remove cytoplasmic residues. Subsequently, the volume of the nuclei was determined and the soluble nuclear content was extracted by treatment with the nuclear lysis buffer (0.01 M HEPES pH 7.9, 0.4 M KCl, 0.0015 M MgCl_2_, 15% (w/v) glycerol, 0.0005 M DTT, 0.0006 M PMSF, 0.0065 mM Leupeptine) for 30 minutes on ice, in combination with repeated vortexing and subsequent centrifugation at 10000 × g (30 min, 4°C). The protein content was determined by the Bradford method using a ready to use solution (BioRad Laboratories GmbH, München, Germany) and bovine serum albumine (BSA) (Merck KGAA, Darmstadt, Germany) as a standard [50]. Concentration of samples and chemical digestion were performed as described above. The copper content was determined using an ICP-MS 820 MS (Bruker Daltonics Inc., Billerica, USA). In case of results below the limit of detection, half this value was used to calculate mean values and standard deviation. Recoveries were determined in the respective matrix using AAS elemental standard solutions (Carl Roth AG) and reached 98% (nuclear fraction) and 102% (cytoplasmic fraction).

### Statistics

Data were analysed for normal distribution and variance was calculated using Levene’s test. Differences in the mean values were determined by using the unifactorial analysis of variance (ANOVA). In case of significance and variance homogeneity post-hoc Dunnett’s-*T* test was applied, in case of inhomogenic variances Dunnett’s-T3 test. Significance testing of two groups was determined by Students’ *t*-test. Software included Valoo 2.4 (analytic-software, Leer, Germany), SPSS 20 (IBM, Armonk, USA) as well as Microsoft Excel 2010 (Microsoft Deutschland GmbH, Unterschleißheim, Germany).

## Abbreviations

AAF: Artificial alveolar fluid; AIF: Apoptosis inducing factor; ALF: Artificial lysosomal fluid; BET: Brunauer-Emmett-Teller analysis; CuCl2: Copper chloride; CuO MP: Copper oxide microparticles; CuO NP: Copper oxide nanoparticles; DMEM: Dulbecco’s Modified Eagle Medium; DMEM/FCS: Dulbecco’s Modified Eagle Medium supplemented with 10% FCS; DLS: Dynamic light scattering; EDX: Energy-dispersive X-ray spectroscopy; EMA: Ethidium monoazide; FCS: Fetal calf serum; GF-AAS: Graphite furnace atomic absorption spectrometry; GSH: Glutathione; H2O: Ultrapure water; ICP-MS: Inductively coupled plasma mass spectrometry; PARP-1: Poly(ADP-ribose)polymerase-1; PBS: Phosphate buffered saline; ROS: Reactive oxygen species; RT: Room temperature; SEM: Scanning electron microscopy; TEM: Transmission electron microscopy; XRD: X-ray diffraction; ZP: Zeta potential.

## Competing interests

The authors declare that they have no competing interests.

## Authors’contribution

AH supervised and coordinated the research project and finalized the manuscript. The study design was performed by AS and AH. AS conducted the majority of the experiments, analysed the data and drafted the manuscript. JO and BW performed part of the experiments and were involved in the particle characterization by DLS. All of the authors have read and approved the final manuscript.
